# Thickness and Substrate Effect on the Mechanical Behaviour of Direct Occlusal Veneers

**DOI:** 10.1016/j.identj.2022.11.006

**Published:** 2022-12-09

**Authors:** João Paulo M. Tribst, Qais Tach, Paul de Kok, Amanda Maria de O. Dal Piva, Ruud H. Kuijs, Cornelis J. Kleverlaan

**Affiliations:** a Department of Oral Regenerative Medicine, Academic Centre for Dentistry Amsterdam (ACTA), Universiteit van Amsterdam and Vrije Universiteit, Amsterdam, The Netherlands; b Department of Dental Materials Science, Academic Centre for Dentistry Amsterdam (ACTA), Universiteit van Amsterdam and Vrije Universiteit, Amsterdam, The Netherlands; c Private practice, Rotterdam, The Netherlands

**Keywords:** Composite resin, Dental veneers, Fixed prosthesis, Finite element analysis

## Abstract

**Purpose:**

This study aimed to evaluate the fracture resistance and stress magnitude of occlusal veneers made of conventional or flowable resin composites at different minimal thicknesses bonded on enamel or dentin.

**Material and methods:**

A total of 120 sound bovine incisors were flattened and used as substrates (enamel or dentin) for the restorations. The teeth were embedded into polymethyl methacrylate and allocated into 4 groups according to the resin composite (Clearfil AP-X PLT and Clearfil Majesty Flow, Kuraray Dental) and substrate. Further, the substrates were randomly subdivided in 12 groups (N = 120, n = 10) according to the occlusal veneer minimal thickness: 0.5, 1.0, or 2.0 mm. The teeth were directly restored with a standardised procedure. Then, the specimens were loaded until fracture in a universal testing machine (Instron 6022, Instron Corp.). A 3-way and a 1-way analysis of variance were used to determine significant differences for each factor. Three-dimensional finite element analysis was carried out following the in vitro boundary conditions to assess the stress magnitude in the restoration during compressive loading.

**Results:**

The fracture loads were recorded into initial load to failure (ILF) and fatal load to failure (FLF). Differences were found in material for ILF and FLF, leading to an overall equal good performance in fracture load and stress distribution for both materials, regardless of the substrate. Differences in thickness were apparent in both ILF and FLF.

**Conclusions:**

Direct conventional and flow resin composite occlusal veneers present a promising mechanical behaviour when bonded on enamel or dentin. However, caution is advised when preparing 0.5-mm minimal thickness restorations.

## Introduction

Loss of tooth tissue by causes other than caries, trauma, or development disorders is a physiologic condition on account of the aging process known as tooth wear.^1^^,^^2^ However, the accelerated loss of enamel and even dentin can be pathologic, resulting in loss of vertical dimension of occlusion and aesthetic problems. This results from a multifactorial aetiology categorised into attrition, abrasion, abfraction, and erosion.^3^ Early diagnosis is essential to perform adequate preventive and therapeutic treatments.^3^^,^^4^ However, due to the slow and asymptomatic process of wear, the loss of vertical dimension of occlusion is often diagnosed at a more advanced stage.^4^ It has been reported that the prevalence of adult patients with severe tooth wear ranges from 3% at the age of 20 years to 17% at the age of 70.^5^

In the initial stages of tooth wear, the treatment in the form of prevention and monitoring may be sufficient; but at a certain point, the restorative therapy will become necessary.^3^^,^^6^^,^^7^ A number of restorative materials have been advocated throughout recent decades, ranging from cast metal restorations to all-ceramic and direct and indirect resin composite restorations, each with their distinct advantages, disadvantages, and restorative strategy.^8^ Furthermore, clinicians need to take patients’ needs, aesthetics, and remaining tooth structure into account.

The longevity of restorations depends upon many different factors such as material, remaining tooth structure, and patient and operator variables.^9^ Due to improvements in adhesive dentistry, a less invasive method of restoring eroded dentitions has become a possible as an alternative to more invasive rehabilitation through extensive full crowns that require a significant amount of dental tissue loss.^10^^,^^11^ Direct resin composites provide survival rates up to 74%, with annual failure rates between 1.5% and 2.2% over 22 years.^11^ In addition, this strategy minimises destruction of sound dental tissue, enabling maintenance of pulp vitality and tissue preservation, therefore preventing tooth strength reduction.^12^ However, the quality of the resin composites are dependent on the skill of the operator and have disadvantages, such as lower wear resistance, bulk fracture, and discolouration.^13^^,^^14^ Nevertheless, recent studies on ultrathin restorations have shown acceptable fatigue resistance and long-term performance with minimal tooth preparation.^15^^,^^16^ However, adeqaute clinical evidence for this approach is still not available. Despite the fact that direct resin composite restorations are now awidespread method to treat abraded or eroded dentition.^16^

Besides the choice of restorative material, restoration thickness has also been reported to affect fracture resistance.^17^ For lithium disilicate, in vitro studies have shown that a reduction of occlusal thickness significantly reduced the fracture resistance of simplified restorations.^18^^,^^19^ Whilst some studies recommend minimal thickness of 1.5 mm, a more recent study has shown that a minimal thickness of 0.5 mm provides comparable results for a specific restoration design.^18^^,^^20^ For resin composites, the same thickness of 1.5 mm has been recommended,^21^ although, there are only a few studies comparing restoration thickness and an acceptable fracture resistance value.

Several studies used simplified restoration to determine appropriate restoration thickness.^22^^,^^23^ Whilst they can elucidate the effect of material properties, the clinical relevance of anatomic restorations is still unclear. Therefore, this in vitro and in silico study evaluated the fracture resistance and mechanical behaviour of anatomic direct composite restorations of different thicknesses, bonded on dentin or enamel. The null hypotheses were as follows: (1) no significant difference would be found in fracture resistance of different resin composites, (2) restoration thickness does not affect fracture resistance, (3) restoring on dentin or enamel does not affect fracture resistance, and (4) the restorations’ mechanical behaviour would be similar regardless of material, minimal thickness, and substrate.

## Material and methods

### Specimen preparation

Two resin composites were used in this study: a conventional high filled hybrid composite and a flowable composite (Table).^24^^,^^25^ A total of 120 sound bovine incisors were used as substrates. The crowns were separated from the roots and subsequently polished at 400 grit to create a flat surface. Half of the crowns were polished until exposing the enamel subsurface and the other half, until the dentin tissue. Thereafter, all the crowns were embedded into polymethyl methacrylate (PMMA; Vertex Dental) with the exposed flat surface parallel to the ground. After curing under pressure (2 bar), the hardened PMMA blocks containing the crowns were removed and polished. After that, they were divided into 12 groups (N = 120, n = 10) according to the composite resin (conventional [CCR], Clearfil AP-X PLT, Kuraray Dental, 86wt%/70vol% filler, or flowable [FCR], Clearfil Majesty Flow, Kuraray Dental, 81wt%/62vol% filler), substrate (enamel or dentin), and restoration minimal thickness (0.5, 1.0, and 2.0 mm).

**Table:** Mean initial (ILF) and fatal (FLF) load to failures in newton (N) and standard deviation (SD) for dentine or enamel substrates, considering restorative material and occlusal minimal thickness.

			Initial load to failure (ILF)			Fatal load to failure (FLF)			Maximum principal stress			Minimum principal stress		
Group	Composite	Substrate	0.5 mm	1.0 mm	2.0 mm	0.5 mm	1.0 mm	2.0 mm	0.5 mm	1.0 mm	2.0 mm	0.5 mm	1.0 mm	2.0 mm
CCR_d_	Conventional	Dentin	1605 (593)^b1^	1664 (790)^b2^	2425 (835)^a1^	2505 (834)^a1^	2884 (1266)^a1^	3048 (813)^a1^	178.22	174.88	175.01	−652	−570.1	−495
FCR^d^	Flowable		1660 (325)^b1^	2382 (354)^a1^	2817 (732)^a1^	2299 (811)^b1^	3126 (770)^a1^	3346 (763)^a1^	178.22	175.12	175.04	−651.8	−569.9	−494.8
CCR^e^	Conventional	Enamel	1696 (839)^b1^	1676 (461)^b2^	2986 (425)^a1^	3077 (736)^a1^	2932 (775)^a2^	3252 (390)^a2^	178.24	175.21	175.07	−652	−561.4	−495.3
FCR^e^	Flowable		1539 (615)^c1^	2131 (297)^b1^	2871 (456)^a1^	2508 (382)^b2^	4184 (421)^a1^	3859 (750)^a1^	178.23	175.16	175.09	−651.9	−570.1	−495.1

Equal letters in each horizontal row and equal numbers in each vertical row (for each substrate) indicate no statistical significant difference.

**Fig. 1:**
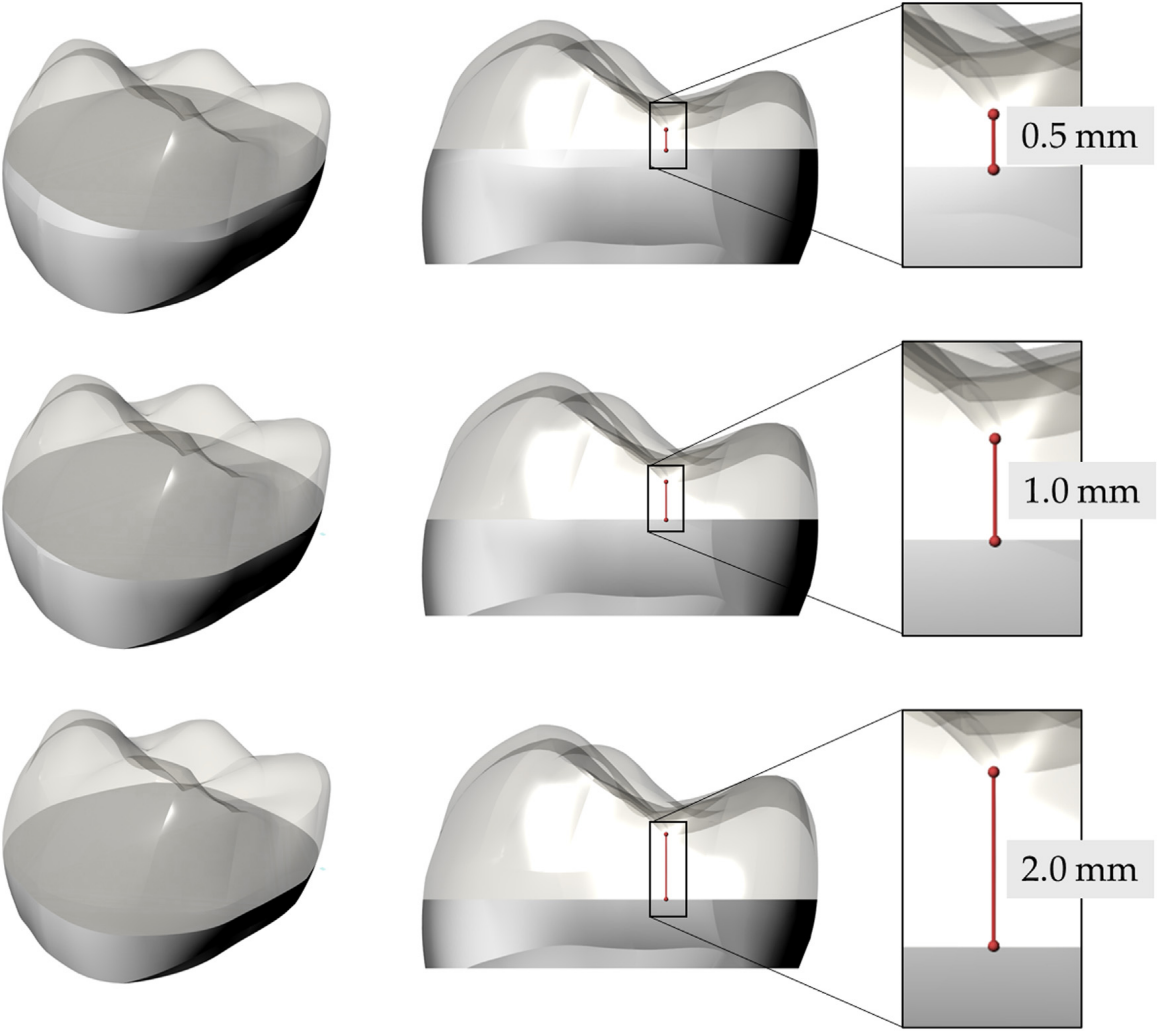
Three different designs of occlusal veneers according to the minimal restoration thickness.

To build standardised occlusal veneers, the one-third occlusal part of a typodont lower first molar was duplicated by making an impression with a light- and heavy-body siloxane impression material (Flexitime, Heraeus Kulzer). The acquired impression was used to make 3 anatomically identical restorations. After polishing, the minimal thicknesses of 0.5, 1.0, and 2.0 mm were obtained and measured with a dental thickness gauge at the deepest point in the central fissure (Figure 1).

Afterwards, an impression was made of these anatomically shaped occlusal veneers with a translucent siloxane impression material (Memosil, Heraeus Kulzer) held together in a plastic ring. The ring and impression material were made thin enough in order to allow the curing light to reach as deep as possible. Small openings were made at the outline of the impression material and plastic ring in order to allow seeping away of excess composite.

For pretreatment of the teeth, they were air-dried, treated with the self-etching primer (Clearfil SE Prime & Bond, Kuraray Dental) for 20 seconds using a microbrush, and air-dried gently for 5 seconds. The bonding agent was applied, gently air-dried, and polymerised for 10 seconds using a blue light (Astralis 10, Ivoclar). The template with the desired thickness was filled with the resin composite and placed on the substrate. A glass plate was used to evenly apply pressure in order to minimise deformation of the restoration. Under finger pressure, the restoration was simultaneously polymerized for 20 seconds. After removing the template, the restoration was again light-cured for 20 seconds in all directions. The final specimens were stored in water at 37 °C for 24 hours for complete polymerization prior to loading.

### Fracture resistance

All specimens were tested in a universal testing machine (Instron 6022, Instron Corp.) with 10-kN load cell and speed of 1 mm/min, and the axial force was applied at the central fissure with a steel ball (12 mm diameter), resulting in contact on the cusps of the restoration. The force applied until fracture resulted in initial fractures (initial load to failure [ILF]) and fatal fractures (fatal load to failure [FLF]). The fracture load values were recorded in newtons.

Afterwards, the composite fragments were evaluated to identify the direction of crack propagation^16^, ^17^, ^18^ with the aid of a stereomicroscope (SZ Olympus). Next, representative specimens were cleaned in an ultrasonic bath with isopropyl alcohol for 10 minutes, dried, gold-sputtered, and analysed with greater magnification using scanning electron microscopy (Evo LS15, Oberkochen, Carl Zeiss) at the acceleration voltage of 10 kV.

### Finite element analysis

To assess the stress distribution for each condition, the 3-dimensional finite element method was applied. For that, an occlusal veneer model was created based in the in vitro specimens and different thickness. A previously reported model^26^ was exported and modified into the computer-aided design software (Rhinoceros version 5.0 SR8, McNeel North America). To allow a homogeneous interface between the restoration and the substrate, the cutplane was used to split the geometries, allowing a perfect adhesive region. This procedure was replicated according to the occlusal veneer thickness (3 ×).

The preprocessing was carried out using FEMAP 11.1.2 (Siemens PLM software), whilst the analysis was done with NX Nastran (Siemens PLM Software). The mesh subdivision was created after meshing each solid structure, considering similar quantity of nodes between the adhesive interface. For all models, parabolic tetrahedron solid elements were used. The material properties have been considered as isotropic, with a Poisson ratio of 0.3. The elastic modulus values were 90 GPa for enamel, 18 GPa for dentin, 16.8^25^ for CCR, and 8.8^26^ for FCR. The contacts were defined based in the coincident nodes, previously merged at the meshing process with bonded behaviour.

The load of 500 N was applied on the cusps,^27^ similar to the in vitro loading. The nodes at the bottom surface of the substrate were selected as the fixation and restrained in all directions. In postprocessing, the contour option “average elemental” without use of the “corner data” was used for visualising the results. The required results were obtained in maximum principal stress and minimal principal stress.

### Statistical analyses

Fracture load data were evaluated by 3-way analysis of variance (ANOVA). In addition, 1-way ANOVA was applied to determine differences for ILF and FLF of each factor separately. The level of significance set at 95% was considered in the statistical software (IBM SPSS Statistics 20) The stress results were evaluated qualitatively using stress maps, and the stress peaks were recorded using the quantitative comparison between different models.

## Results

### Fracture resistance

**Fig. 2:**
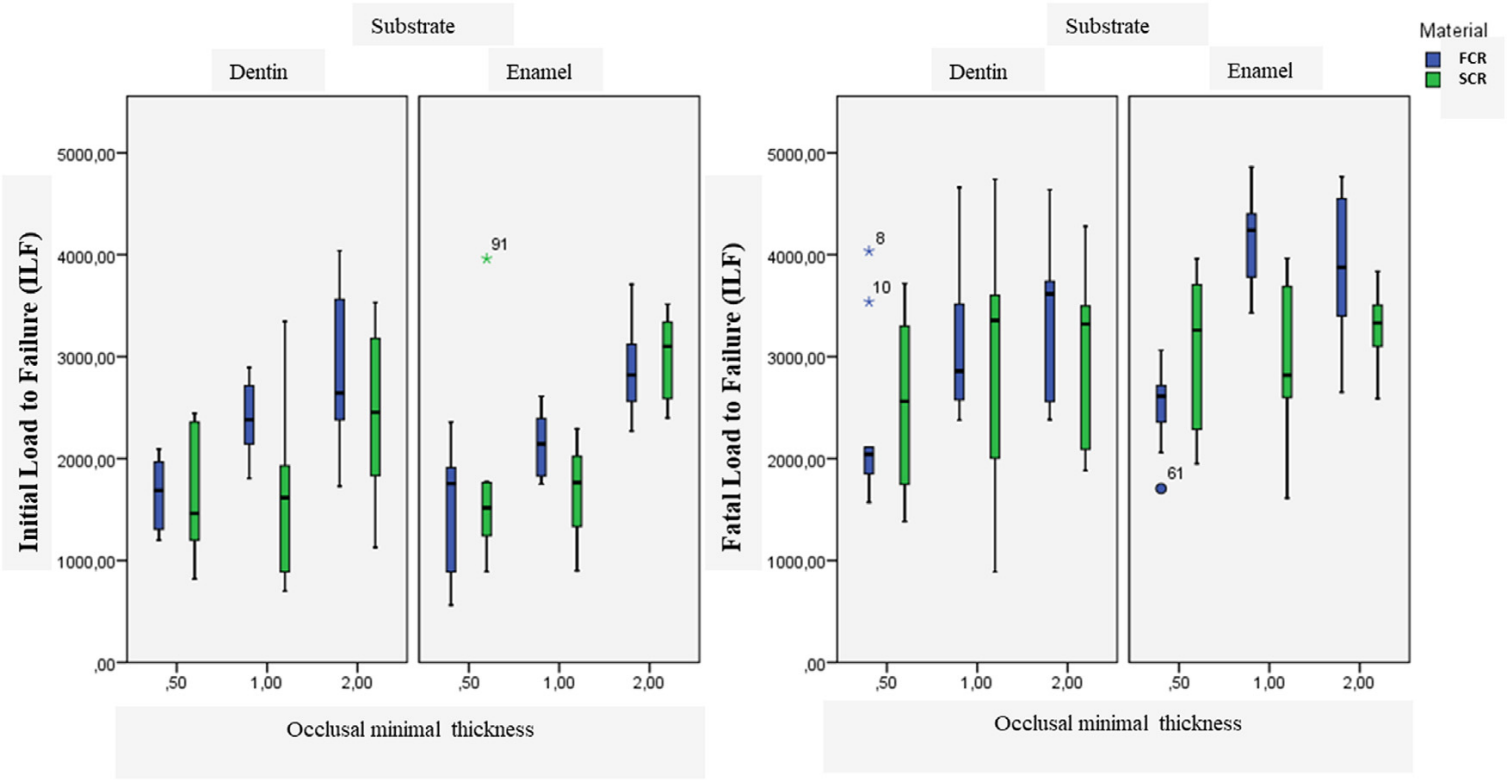
Mean initial (ILF) and fatal (FLF) load to failures in Newton according to the occlusal minimal thickness.

After the compressive test, 28.3% of the failed specimens exhibited fatal fracture. The other specimens exhibited 1 or more cracks prior to fatal fracture. This resulted in the division of the fracture loads results into ILF and FLF. One specimen bonded to enamel (CCR with 2.0 mm) was excluded because of unclear load values. The results of the load tests are shown in the Table and in Figure 2.

For IFL, 3-way ANOVA revealed that composite resin (*P* = .042, *F* = 4.2) and occlusal minimum thickness (*P* < .001, *F* = 38.9) influenced on the load to failure, whilst substrate (*P* = 0.599, *F* = 0.3) did not. This was different for FLF, where substrate (*P* = .003, *F* = 9.6) and occlusal minimum thickness (*P* < .001, *F* = 12.3) affected on the load to failure, whilst the composite resin did not (*P* = .056, *F* = 3.7). In addition, results show that FLF loads were higher than ILF loads.

**Fig. 3:**
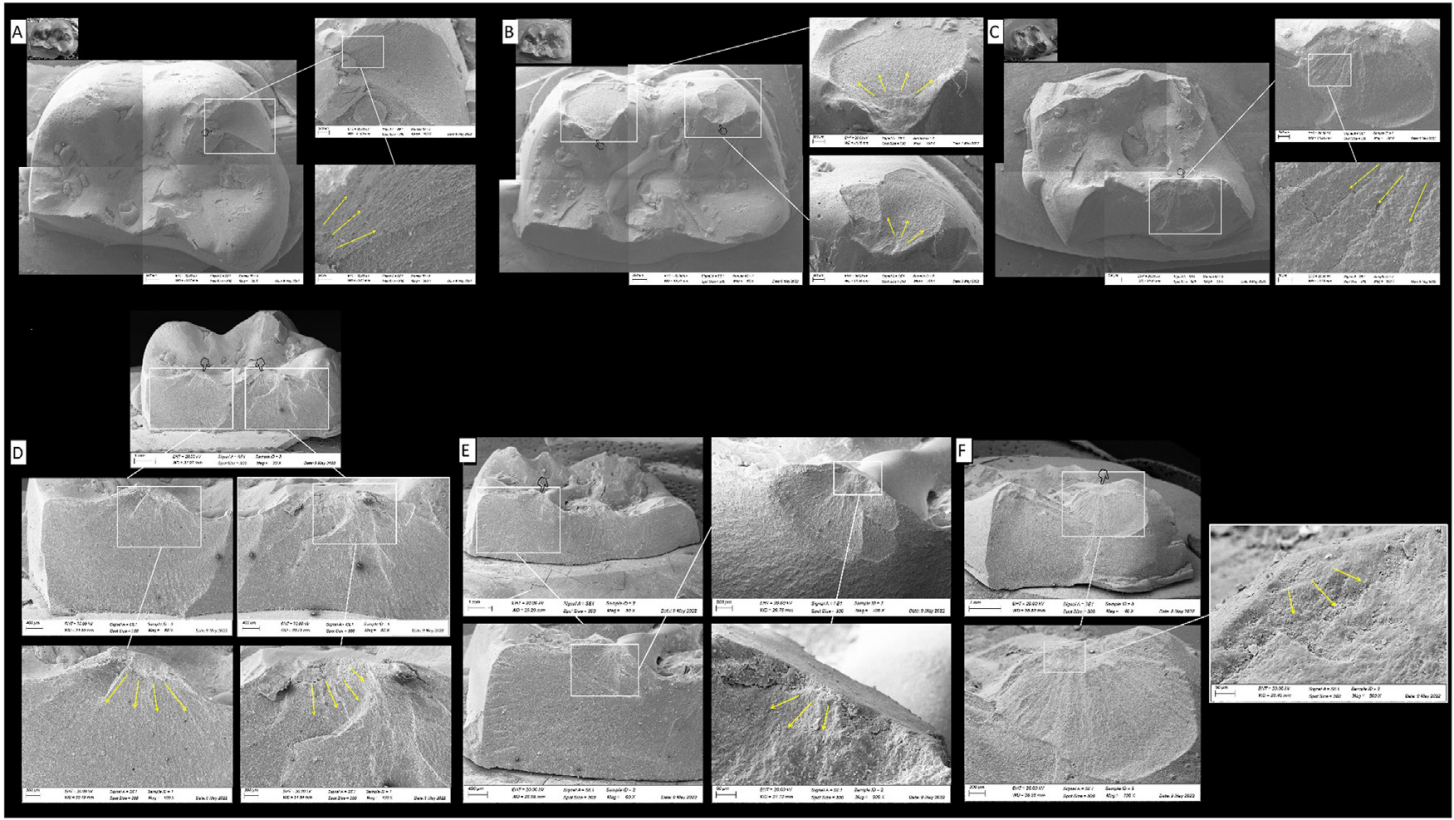
Scanning electron microscopy representative fatal failed specimens with 2.0 mm (A), 1.0 mm (B), and 0.5 mm (C) thickness in occlusal view and 2.0 mm (D), 1.0 mm (E), and 0.5 mm (F) thickness in lateral view. The images show the largest fragment with deformed areas at the contact point (pointer) and the direction of crack propagation (yellow arrows) from occlusal to the cervical.

The failure origin could not be determined due to the continuous effect of compressive loading with overlapping fracture features (Figure 3). However, the direction of crack propagation could be identified, showing that the crack started at the indenter contact points to the cervical, with visible damage in more than 1 cusp and several cracks.

### Finite element analysis

**Fig. 4:**
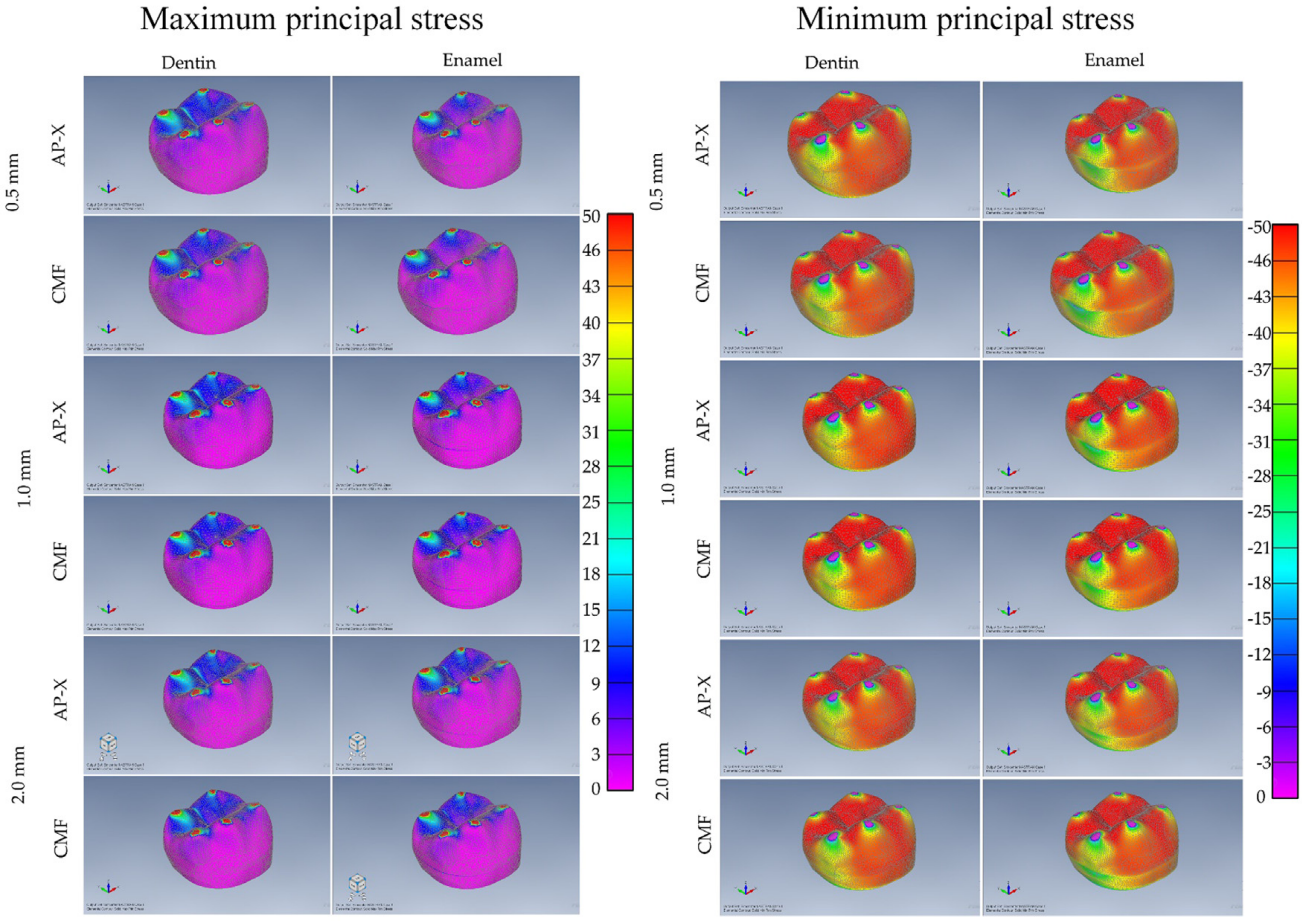
Maximum principal and minimum principal stress results in the occlusal veneer according to the substrate, composite resin, and restoration thickness.

The numerical stress analysis results are summarised in Figure 4. The positive values represents tensile stresses, whilst the negative values represent the amount of compressive stress, both concentrated in the contact points on top of the cusps. Therefore, the results showed similar maximum and minimal stress regions for occlusal veneer restorations.

Observing the tensile stress distribution, there is a similar stress pattern between the models for the mechanical response in the cusps. However, thin restorations bonded on dentin showed larger areas of stress distribution, extending from the cusps and involving the secondary sulcus. However, for the compressive stress, there is a qualitative difference between the restorations. The margin of the restoration is affected by the restorative material, restoration thickness, and substrate.

All models exhibited the highest stress values in the regions in red from the stress maps. Despite the slight differences for the criteria, it is noticeable that in compressive loading on the cusps tips, subjected to the first contact with the antagonist, the restorations will fail first due to the high stress magnitude. Tensile stress peaks analysis revealed, for both substrates, similarity between composite resin veneers. The stress peaks of the models are summarised in the Table.

## Discussion

This study investigated the fracture resistance of occlusal veneers in 2 resin composites at 3 different minimal thicknesses, bonded to dentin or enamel. Within the limitations of this study, the null hypotheses that no difference would be found in fracture resistance considering (1) material type, (2) restoration thickness, and (3) substrate were partially rejected.

Results revealed that in a specific situation (ie, restoring on enamel), the choice of restorative material does significantly influence fracture resistance. But in several other situations, as shown in Figure 2, the choice of restorative material does not influence the fracture resistance. Special attention must be provided for FCR at 0.5 mm bonded to enamel, achieving the lowest initial load to start a failure. For dentin, similar behaviour was observed for 0.5 and 2 mm, whilst at 1 mm, the FCR presented higher ILF.

In addition to the fact that both resin composites have similar filler content, CCR has approximately 29% higher flexural strength (204 MPa) than FCR (145 MPa). This suggests that CCR should have higher fracture resistance than FCR. However, at a 1-mm thickness, FCR showed higher ILF for both substrates and also for enamel considering FLF. This can be explained by the significant lower elastic modulus of FCR (8.8 GPa) because the development of tensile stresses is more related to the elastic modulus than other material properties.^16^ To evaluate the clinical performance of such FCR over longer periods of time, long-term clinical studies are required.^28^^,^^29^

When restoring enamel or dentin with CCR, the thickness does not influence the FLF. However, the ILF seems to be affected by the differences in the material thickness, partially rejecting the hypothesis that restoration thickness does not influence fracture resistance. A preliminary review recommended a ceramic material thickness between 0.7 and 1.0 mm^29^; in addition, thicknesses less than 0.7 mm are recommended to be used in resin-based materials for occlusal veneers. The present study corroborates these statements because groups with a 0.5-mm thickness showed lower mean values that were statistically similar to 1 mm. Another study investigated occlusal veneers in 1.0 and 1.5 mm made in lithium disilicate, hybrid ceramic, and nanoceramic resin.^30^ The authors observed that all conditions achieved loads higher than the normal bite force and that resin composite veneers showed the best failure pattern.

This study did not aim to directly compare enamel and dentin. However, it is important to emphasise that in almost all evaluated conditions, restoring on dentin or enamel did not influence fracture resistance. These findings could be considered remarkable taking into account the relatively low elastic modulus of these restorative materials. Both are close to the elastic modulus of dentin (18 GPa), whilst the elastic modulus of 90 GPa of enamel is considerably higher.^31^^,^^32^ Such a discrepancy in elasticity allows an earlier radial crack formation, increasing the composite resin failure risk.^33^ The present findings contradict the abovementioned mechanisms, suggesting that factors other than the elastic modulus can influence the mechanical response. However, this is only limited to mechanical load and wear mechanisms, such as fatigue wear, which can influence the material long-term stability. In addition, different mechanical behaviour has been observed in the colourimetric graphs for both maximum and minimum principal stress criteria, rejecting hypothesis 4. Thinner veneer on dentin showed higher stress concentration areas; while minimum principal stress criteria showed different pattern for the restoration margin.

A previous study evaluated the fracture load of lithium disilicate occlusal veneers luted to enamel or dentin, and similar to the present study, the authors did not find a difference between substrates.^34^ Another investigation with anatomic specimens compared the fracture load of lithium disilicate, zirconia, or polymer-infiltrated ceramic occlusal veneers bonded in enamel or dentin.^35^ No differences were found between substrates. Different from both reported studies,^34^^,^^35^ the present investigation complements the in vitro result with the stress analysis. The results of stress are usually correlated with the failure origin. In this scenario, the results show not only a similar stress trend between the groups but also that the contact region is the most stressed area in this loading method. Therefore, the importance of the loading region and occlusal contact points for the fracture and stress distribution was demonstrated. Occlusal contacts have a substantial influence on the positioning of teeth to maintain the position and stability of the mandible. Axial loads should be able to generate more uniform stresses.^36^

Fracture load associated with finite element analysis was used to evaluate the influence of material and thickness on reliability and stress distribution of occlusal veneers.^37^ There were competitive failure modes originating from the occlusal indentation area and radial cracking from the intaglio surface for their fractured specimens. The present study confirms these findings, showing similar fracture features, but with the limitation of absence of a fatigue effect. In addition, the preliminary study^37^ collected maximum and minimal principal stresses, demonstrating that thinner restoration models accumulated more stress in the structures than thicker restorations models. This can be justified by the loading method, which was different from the present study considering only a single region.

This study tried to mimic the clinical severe tooth wear conditions as much as possible; nonetheless, several limitations were present. The direct restoration using a mould reduced the handling control and allowed possible inclusion of voids or defects. Furthermore, bovine teeth were used, which limits interpretation.^31^ Nevertheless, several studies concluded that bovine teeth are an acceptable surrogate, keeping the differences in mind.^32^ Additionally, this study is limited to single loading, not taking into account the effect that other external factors in the oral cavity have on material properties.^23^^,^^38^ Other studies showed that fatigue testing, mimicking human chewing forces, influences the performance of resin composites^39^^,^^40^ as well as water sorption, ageing, and wear.^41^ However, fracture resistance in nearly all studies was high enough, withstanding maximum human masticatory forces of 900 N.^42^ Further research could include artificial ageing and fatigue testing with the same restoring procedure and possibly including ceramics to compare and mimic the clinical situation.

## Conclusions

Within the limitations of this study, both resin composites perform equally well. Furthermore, restoring on dentin or enamel does not influence fracture resistance or the restoration stresses. Nonetheless, direct resin composite occlusal veneers show acceptable fracture resistance, as nearly all experimental groups exceed human maximum masticatory forces.

## Author contributions

João Paulo M. Tribst: conception and design, acquisition of data, analysis and interpretation of data, drafting the article, and final approval. Qais Tach: conception and design, acquisition of data, drafting the article, and final approval. Paul de Kok: conception and design, acquisition of data, drafting the article, and final approval. Amanda Maria de O. Dal Piva: acquisition of data, drafting the article, revising the article, and final approval. Ruud H. Kuijs: analysis and interpretation of data, drafting the article, revising the article, and final approval. Cornelis J. Kleverlaan: analysis and interpretation of data, drafting the article, revising the article, and final approval.

## Conflict of interest

None disclosed.
